# Validation of the “Patient‐Acceptable Symptom State” Question as Outcome Measure in AChR Myasthenia Gravis: A Multicentre, Prospective Study

**DOI:** 10.1111/ene.70262

**Published:** 2025-06-25

**Authors:** Gregorio Spagni, Massimiliano Ugo Verza, Sara Cornacchini, Francesca Beretta, Bo Sun, Antonio Lotti, Silvia Falso, Alessandro Barilaro, Luca Massacesi, Amelia Evoli, Valentina Damato

**Affiliations:** ^1^ Department of Neurosciences, Psychology, Drug Research and Child Health (NEUROFARBA) University of Florence Florence Italy; ^2^ Department of Neurosciences and Sensory Organs Careggi University Hospital Florence Italy; ^3^ Nuffield Department of Clinical Neurosciences University of Oxford Oxford UK; ^4^ Department of Neurosciences Catholic University of the Sacred Hearth Rome Italy

**Keywords:** myasthenia gravis, ocular symptoms, outcome, patient‐acceptable symptom state, patient‐reported outcome measures, quality of life

## Abstract

**Introduction:**

Patient Acceptable Symptom State (PASS) is emerging as a valuable subjective measure of the overall myasthenia gravis (MG)‐related burden. This study aimed at identifying PASS‐positive thresholds for the most used clinical scales, investigating whether PASS and MGFA post‐intervention status capture different aspects of the disease outcome, and identifying clinical variables associated with PASS=YES response.

**Methods:**

Adult AChR‐MG patients were prospectively enrolled at two Italian Centres (Rome: index cohort; Florence: validation cohort). PASS thresholds for MG‐ADL, QMG, and MG‐QOL15r were defined in the index cohort by ROC analysis and validated in the validation cohort; predictors of favorable PASS were identified by multivariable analysis.

**Results:**

This study included 173 patients (44% females, median age at onset: 53 years). PASS=YES patients had significantly lower median MG‐ADL, QMG, and MG‐QOL15r scores, with the following thresholds for PASS=YES: MG ADL ≤ 2, QMG ≤ 8 and MG‐QOL15r ≤ 6. The MG‐ADL (OR = 0.46, 95% CI = 0.36–0.60, *p* < 0.001), QMG (OR = 0.72, 95% CI = 0.64–0.81, *p* < 0.001) and MG‐QOL15r (OR = 0.76, 95% CI = 0.70–0.84, *p* < 0.001), were independently associated with a favorable PASS. The degree of ocular involvement in each scale was the strongest negative determinant of PASS=YES.

**Conclusions:**

This study validates the PASS question and highlights the relevance of ocular complaints in patients' perception of MG burden.

## Graphical abstract

The aim of this study was to further validate the patient‐acceptable symptom state (PASS) question in myasthenia gravis (MG), establish threshold scores on commonly used clinical scales that indicate whether a patient considers their current MG control to be acceptable (PASS = YES) and analyse its main clinical predictors. The study included 173 adult AChR‐MG patients and identified the following PASS‐positive cut‐offs: MG‐ADL ≤2, QMG ≤8 and MG‐QOL15r ≤6. These findings were validated across two independent cohorts. Lower scores on these scales were significantly associated with a favourable PASS. Furthermore, ocular symptoms emerged as the most significant negative factor influencing patients’ perception of well‐being, highlighting their substantial impact on the overall disease burden.

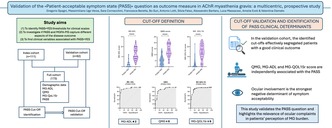

## 1 Introduction

The treatment of Myasthenia Gravis (MG) has recently witnessed a significant improvement with the approval of targeted therapies for patients with acetylcholine receptor (AChR) antibody‐positive MG (AChR‐MG) [1, 2, 3]. Despite these advancements, a proportion of these patients do not achieve a satisfactory control of their disease [4]. In this context, the inclusion of the patient perspective in the outcome evaluation is now considered crucial to capturing significant improvement. The use of the Patient Acceptable Symptom State (PASS) question is emerging as a valuable measure to provide insights into the overall burden of MG [5, 6, 7]. However, PASS question thresholds for the most used clinical scales, such as the Myasthenia Gravis Activities of Daily Living (MG‐ADL), the Quantitative Myasthenia Gravis (QMG) score, and the MG Quality of Life‐15 (MG‐QOL15r) have only been indirectly derived [5]. In addition, factors associated with a favorable PASS, and whether it captures a different dimension of clinical outcome compared to the Myasthenia Gravis Foundation of America (MGFA) post‐intervention status (PIS) have not been investigated.

In this study we aimed at (1) identifying PASS‐positive thresholds for the most used clinical scales and validating the PASS question in AChR‐MG; (2) investigating whether PASS and MGFA‐PIS capture different aspects of the disease outcome; (3) identifying which clinical variables are independently associated with a favorable PASS response in a prospective, multicentre cohort of AChR‐MG patients.

## 2 Methods

This study was performed from January 2021 to January 2024 at two Italian reference Centres for MG, Fondazione Policlinico Universitario “A. Gemelli” IRCCS (FPG), Rome (index cohort; enrolment: 2021–2022), and Azienda Ospedaliero‐Universitaria Careggi (AOUC), Florence (validation cohort; enrolment: 2022–2024), and included consecutive patients fulfilling the following criteria: (a) diagnosis of AChR‐MG [8], (b) age ≥ 18 years at enrolment, (c) written informed consent. Enrolment was limited to AChR‐MG patients to ensure the inclusion of a pathophysiologically homogeneous population. The PASS question was asked to all the patients included in the study (see Appendix). Two independent evaluators translated the original PASS question into Italian, which was independently reviewed and pre‐tested on a small MG patient sample and then refined for clarity and cultural appropriateness (more details in the Supplementary Methods). Patients were evaluated with the QMG [9], MG‐ADL [10], and MG‐QOL15r [11]. This study was approved by the two Institutions (protocol n. 22914_bio, approved by AOUC; E.C. protocol number 0008363/21, approved by FPG), and was conducted according to the Helsinki Declaration. All patients provided written consent to participate in this study.

### 2.1 Statistical Analysis

Receiver operating characteristic (ROC) curves were used to estimate the thresholds of the QMG, MG‐ADL, and MG‐QoL15r scores associated with a favorable PASS. Mann–Whitney and *χ* ^2^ tests were used to compare continuous and categorical variables, respectively (normality test used: Shapiro–Wilk). Clinically significant variables were included in the multivariable logistic regression models used to identify covariates independently associated with PASS=YES (see Supplementary Methods). The agreement between PASS=YES and MGFA‐PIS = MM‐or‐better was analyzed with Cohen's K. Statistical analysis was performed with STATA v.18 (STATA Corp, TX, USA) and GraphPad Prism v10 (GraphPad Software, CA, USA).

## 3 Results

This study included 173 patients, 76 (44%) females, with a median age at onset of 53 years (IQR = 30–66), 124 (72%) reporting an acceptable symptom state (PASS=YES) and 49 (28%) reporting an unacceptable symptom state (PASS=NO); 111 patients were recruited at FPG, Rome (index cohort) and 62 at AOUC, Florence (validation cohort) (Table S1).

### 3.1 PASS Validation and Identification of PASS Thresholds

**FIGURE 1:**
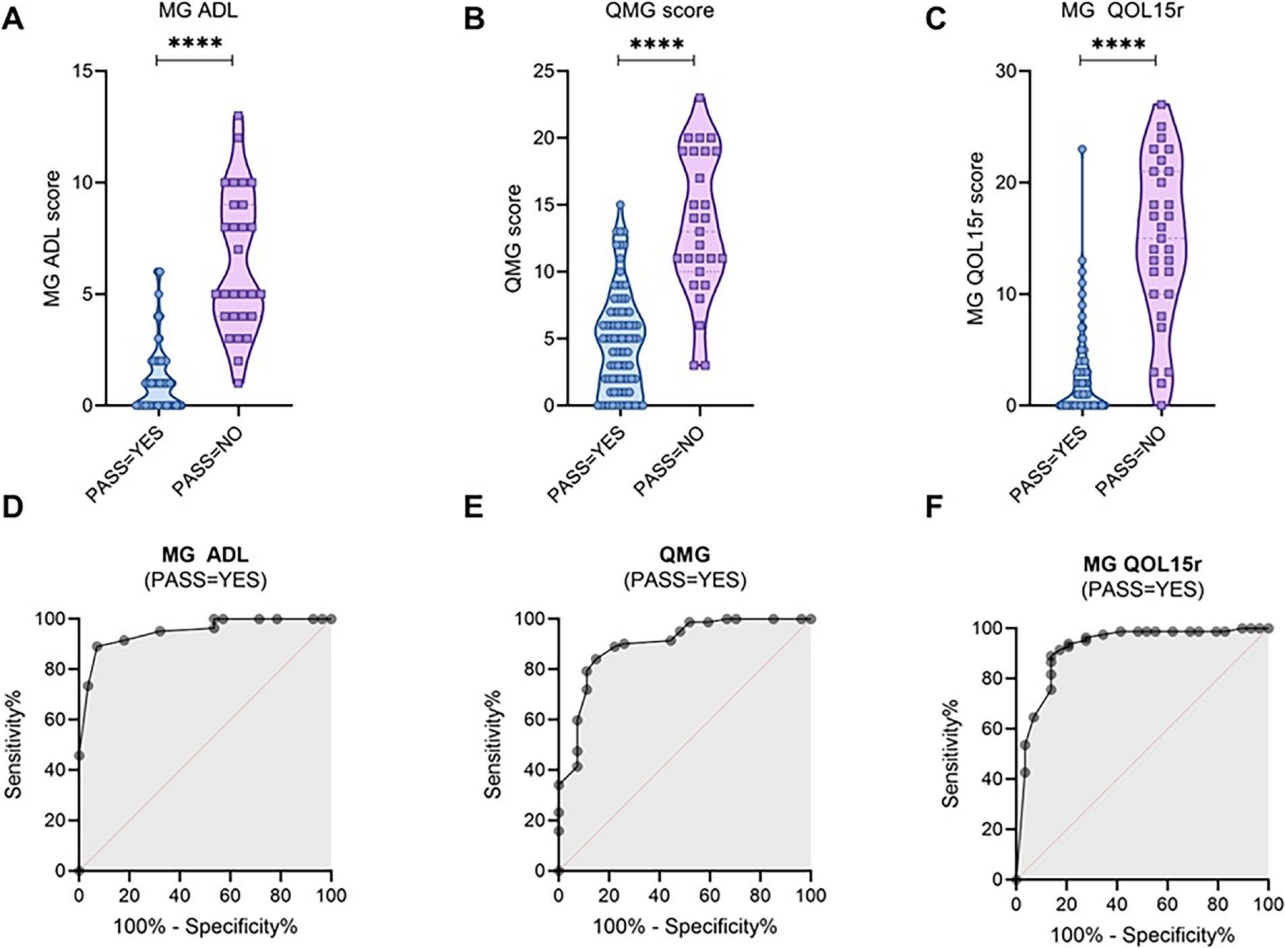
MG clinical scale scores and reply to the patient acceptable symptom state (PASS) question. PASS = YES patients had a lower median MG‐ADL score (1 vs 5, *p* < 0.0001) (A), QMG score (4 vs 11, *p* < 0.0001) (B), and MG‐QOL15r score (1 vs 12.5, *p* < 0.0001) (C) compared to PASS = NO patients of the index cohort. Receiver operating characteristic (ROC) curve estimating the PASS threshold for MG‐ADL score (AUC = 0.92, 95% CI 0.86–0.97) (D), QMG score (AUC = 0.85, 95% CI 0.77–0.93) (E), and MG‐QOL15r score (AUC = 0.92, 95% CI 0.80–0.95) (F) of the index cohort. MG‐ADL, Myasthenia gravis activity of daily living score; MG‐QOL15r, myasthenia gravis quality of life 15 revised score; PASS, patient acceptable symptom state; QMG, quantitative myasthenia gravis score.

In the index cohort, both PASS=YES and MGFA‐PIS = MM‐or‐better were associated with lower MG‐ADL, QMG, and MG‐QOL15r scores (*p* < 0.0001, Table 1; Figure 1A–C; Figure S1A–C). Based on the ROC analysis (Figure 1D–F; Figure S1D–F), the MG clinical scales' thresholds to best discriminate between patients with and without favorable PASS were: MG ADL ≤ 2, QMG score ≤ 8, and MG‐QOL15r ≤ 6 (Table S2; thresholds for MGFA = MM‐or‐better are also reported in the table).

**TABLE 1:** Univariate and multivariable analysis of demographic and clinical factors associated with achievement of favorable outcome defined by the “patient acceptable symptom state” (PASS) question.

	PASS = YES (*N* = 124)	PASS = NO (*N* = 49)	*p*
Median age at onset, IQR (years)	57.5 (36–67)	45 (26–63)	**0.037**
Female, *n* (%)	50 (40%)	26 (53%)	0.13
Median disease duration, IQR (years)	6.7 (3–14.3)	6.4 (1–14)	0.40
*MG subtype*			
EOMG	35 (28%)	16 (33%)	0.583
LOMG	22 (18%)	2 (4%)	**0.019**
VLOMG	30 (24%)	9 (18%)	0.409
Thymoma‐associated MG	37 (30%)	22 (45%)	0.06
*Maximum MGFA clinical classification*			
I	18 (15%)	4 (8%)	0.318
II	44 (36%)	9 (18%)	**0.029**
III	33 (27%)	26 (53%)	**0.0013**
IV	14 (11%)	6 (12%)	0.99
V	15 (12%)	4 (8%)	0.59
Moderate MG to MG crisis (MGFA: III‐V) at maximum disease severity	62 (50%)	36 (74%)	**0.0063**
Bulbar involvement at max. disease severity, *n* (%)	69 (56%)	30 (61%)	0.504
*MG clinical scales*			
Median MG‐ADL score, IQR	1 (0–2)	5 (3–8.5)	**< 0.0001**
Median QMG score, IQR	4 (2–6.5)	11 (8–15)	**< 0.0001**
Median MG‐QOL15r score, IQR	1 (0–3)	12.5 (5.5–19.5)	**< 0.0001**
Comorbidities	94 (80%)	38 (83%)	0.669
*Treatment*			
No IS	22 (18%)	10 (20%)	0.66
CS	45 (36%)	15 (31%)	0.59
CS + 1 IS	45 (36%)	14 (29%)	0.377
CS + 2 or more IS	12 (10%)	10 (20%)	0.0752
Treatment‐related AEs	47 (38%)	20 (41%)	0.723
*MGFA PIS* ^*a*^			
CSR	14 (11%)	0	**0.0114**
PR	14 (11%)	0	**0.0114**
MM	78 (63%)	7 (14%)	**< 0.0001**
I	13 (10%)	20 (41%)	**0.0004**
U	1 (1%)	4 (8%)	**0.0231**
W	3 (2%)	9 (18%)	**0.0007**
MM‐or‐better	105 (85%)	7 (17%)	**< 0.0001**

*Note:* The table shows the association of the demographic and clinical characteristics with the PASS status, using the univariate. Significant *p* values are showed in bold. a: not applicable in 10 patients (*n* = 1 PASS yes, *n* = 9 PASS no) as the study visit occurred before therapy initiation.

Abbreviations: AE, adverse event; C.I., confidence interval; CS, corticosteroids; CSR, complete stable remission; EOMG, early‐onset myasthenia gravis; I, improved; IQR, interquartile range; IS, immunosuppressant; MG, myasthenia gravis; MG‐ADL, myasthenia gravis activity of daily living scale; MGFA, Myasthenia Gravis Foundation of America; MG‐QOL15r, myasthenia gravis quality of life 15‐revised; MM, minimal manifestations; PASS, patient‐acceptable symptom state; PR, pharmacological remission; QMG, quantitative myasthenia gravis scale; TAMG, thymoma‐associated myasthenia gravis; U, unchanged; VLOMG, very‐late onset myasthenia gravis; W, worsened.

By applying the thresholds of each scale to the validation cohort, all cut‐offs reliably identified patients with a favorable outcome, as defined by an MGFA‐PIS = MM‐or‐better and/or a positive response to the PASS question (Table S3). When the ROC analysis was applied to the validation cohort to identify the best MG scales' thresholds for PASS=YES, the cut‐off values of the index cohort were confirmed only for the MG‐ADL score (≤ 2, sensitivity 75.6%, specificity 68.4%), while lower cut‐off values were identified for the QMG (≤ 6, sensitivity 80.9%, specificity 80%) and the MG‐QOL15r (≤ 3, sensitivity 80.6%, specificity 80%).

### 3.2 Clinical Determinants of the PASS

Clinical determinants of PASS status were analyzed in the total patient cohort. Older age at disease onset, milder MG severity, lower MG‐ADL, QMG, and MG‐QOL15r scores at study visit, and the frequency of MGFA‐PIS = MM‐or‐better were associated with PASS=YES (Table 1). In line with these findings, the MGFA‐PIS = MM‐or‐better and the PASS status showed substantial agreement (Cohen K = 0.63, standard error: 0.08; *p* < 0.0001), although the two measures were discordant in almost 15% of the cases (MGFA‐PIS of MM‐or‐better in 7/41 of PASS=NO and MGFA‐PIS other than MM‐or‐better in 18/123 of PASS=YES patients, *p* = 0.8). A moderate correlation between MG‐ADL and QMG (*r_s_* = 0.79, *p* < 0.0001), MG‐ADL and MG‐QOL15r (*r_s_* = 0.76, *p* < 0.0001), and QMG and MG‐QOL15r (*r_s_* = 0.66, *p* < 0.0001) was found. VIF values confirmed moderate collinearity (MG‐ADL: 2.85; QMG: 2.44; MG‐QOL15r: 1.65). To ensure a more conservative approach and minimize the risk of overfitting, separate models were built for each clinical scale, showing that the MG‐ADL (OR = 0.46 for 1‐point increase, 95% CI = 0.36–0.60, *p* < 0.001), QMG (OR = 0.72 for 1‐point increase, 95% CI = 0.64–0.81, *p* < 0.001) and MG‐QOL15r score (OR = 0.76, 95% CI 0.70–0.84, *p* < 0.001) were independently associated with a favorable PASS, while controlling for MG subtype, maximum MG severity, disease duration, and treatment burden (Table S4). AUC comparison showed no significant differences between the three models in predicting PASS=YES (*p* = 0.419, DeLong Test).

Next, we evaluated whether the muscle district involvement (ocular, limb or bulbar), as assessed by each clinical scale, had an influence on the PASS answer. By multivariable logistic regression, we found that the ocular item subgroup of the MG‐ADL (OR = 0.46 for 1‐point increase, 95% C.I. 0.31–0.67, *p* < 0.001), the QMG (OR = 0.65 for 1‐point increase, 95% C.I. 0.51–0.84, *p* = 0.001), and the MG‐QOL15r (OR = 0.29 for 1‐point increase, 95% C.I. 0.13–0.67, *p* = 0.003) were negatively associated with PASS=YES, while adjusting for the degree of involvement of the limb and bulbar districts (Table S5).

Lastly, we investigated whether, within the same PASS subgroup (PASS=YES or PASS=NO), age at onset, sex, and maximum MG severity correlated with the degree of clinical severity (QMG score), independence (MG‐ADL) and MG‐related QoL (MG‐QOL15r) recorded at the study visit. We limited this analysis to generalized MG, as ocular MG patients have lower QMG, MG‐ADL, and MG‐QOL15r values due to the limited disease expression. The results are reported in Table S6. This analysis showed that, within the PASS=YES subgroup, patients with a history of a more severe disease (maximum MGFA = III to V) had a significantly higher median QMG score compared to those with mild MG at disease peak [5 (IQR = 3–8) vs. 4 (IQR = 2–6), *p* = 0.022]; among PASS=NO patients, those with a milder disease history had a significantly lower median QMG [7 (IQR = 2–11) vs. 12 (IQR = 10–19), *p* = 0.008] and MG‐ADL scores [3.5 (IQR = 2–4.5) vs. 6.5 (IQR = 4–9), *p* = 0.0115] compared to those with a maximum MGFA = III‐V. Subgroups based on age at onset (< or ≥ 50 years) and sex showed no significant differences.

## 4 Discussion

In this study we validated the PASS question as an effective, concise tool to assess AChR‐MG patients' satisfaction with their disease control. Similar to what reported before [5], in our study the patients who responded positively to the PASS question had a significantly lower disease severity. In addition, we directly calculated the acceptable symptom state (PASS=YES) thresholds for MG‐ADL, QMG and MG‐QOL15r scales, validating them in an independent cohort. Notably, the same thresholds of the QMG and MG‐ADL scores for MM‐or‐better found in this study were recently identified in a large Japanese cohort, which likely reflects the objectivity of the MGFA‐PIS and of these scales [12]. In our study, patients who experienced a severe disease course perceived as acceptable a disease burden higher than in mildly affected patients. This suggests that different PASS‐positivity cut‐offs may apply to patient subgroups and that patients' resilience and expectations regarding disease control influence the perceived disease burden. This finding may also partially explain why the optimal thresholds defining PASS‐positive patients in the index cohort differed to some extent from those of the validation cohort, which was characterized by a higher frequency of mild generalized MG.

Our study revealed that the three most commonly used MG clinical scales performed equally in detecting an improvement relevant from the patient perspective. On the other hand, in a proportion of cases, the MGFA‐PIS of MM‐or‐better was not associated with a positive PASS, suggesting that these outcome measures capture different dimensions of the disease. Moreover, we found that the involvement of different muscular districts contributes to the perceived disease burden, with ocular complaints having the strongest negative impact on patient satisfaction. From our findings, ocular complaints should be taken more into account in the design of future studies, considering that purely ocular MG patients have been so far excluded from randomized‐controlled trials (RCTs) [13, 14].

This study has several limitations, such as the inclusion of only AChR antibody‐positive patients, its cross‐sectional nature, the frequency of missing data in some analyses, and the lack of systematic assessment of potential confounders of the PASS response, like comorbid depression [15] or employment status [5].

In summary, this study validated the PASS question as a helpful, reliable, and non‐redundant patient‐reported outcome measure, which easily enables the incorporation of the patient perspective in the MG outcome evaluation. This assessment should be included in current clinical practice and in future RCTs, as it can unveil unmet clinical needs and contribute to the improved care of patients with MG.

## Author Contributions

**Gregorio Spagni:** conceptualization, investigation, formal analysis, visualization, writing – original draft, writing – review and editing, methodology, data curation. **Massimiliano Ugo Verza:** data curation, investigation, writing – original draft. **Sara Cornacchini:** data curation, investigation. **Francesca Beretta:** data curation, investigation, writing – review and editing. **Bo Sun:** formal analysis, writing – review and editing. **Antonio Lotti:** investigation, data curation. **Silvia Falso:** data curation, investigation. **Alessandro Barilaro:** data curation, investigation. **Luca Massacesi:** writing – review and editing, methodology, resources. **Amelia Evoli:** conceptualization, writing – review and editing, resources, supervision, data curation, investigation. **Valentina Damato:** conceptualization, methodology, data curation, investigation, supervision, funding acquisition, writing – review and editing, writing – original draft, project administration, resources.

## Conflicts of Interest

The authors declare no conflicts of interest.

## Supporting information


**Figure S1.** MG clinical scale scores and MGFA‐PIS. In the index cohort, patients with an MGFA‐PIS = MM‐or‐better had a lower median MG‐ADL score [0 (IQR = 0–1) vs. 5 (IQR = 2–8), *p* < 0.0001] (A), QMG score (4 (IQR = 1–6) vs. 11 (IQR = 9–15), *p* < 0.0001) (B), and MG‐QoL15r score (1 (IQR = 0–4) vs. 12.5 (IQR = 2–20.5), *p* < 0.0001) (C) compared to patients who did not achieve MM‐or‐better. Receiver operating characteristic (ROC) curve estimating the MGFA‐PIS = MM‐or‐better thresholds for MG‐ADL score (AUC = 0.91, 95% C.I. 0.86–0.97) (D), QMG score (AUC = 0.95, 95% C.I. 0.90–0.99) (E), and MG‐QoL15r score (AUC = 0.82, 95% C.I. 0.73–0.91) (F) in the index cohort. MG‐ADL = Myasthenia gravis activity of daily living score; MG‐QoL15r = myasthenia gravis quality of life 15 revised score; PASS = patient acceptable symptom state; QMG = quantitative myasthenia gravis score.

**Table S1.** Comparison of the patient characteristics from the index and validation cohort.
**Table S2.** Threshold of MG clinical scales for the definition of favorable outcome according to the PASS and MGFA‐PIS identified in the index cohort.
**Table S3.** Validation of the MG scales defined patient acceptable symptom state (PASS) thresholds in validation cohort.
**Table S4.** Multivariable logistic regression models to evaluate the clinical predictors of PASS=YES.
**Table S5.** MG scales items association with patient acceptable symptom state (PASS) = YES: multivariable logistic regression.
**Table S6** Analysis of the association of demographic and clinical factors with disease expression at study visit within the same PASS‐defined patient subgroup.

## Data Availability

The data that support the findings of this study are available on request from the corresponding author. The data are not publicly available due to privacy or ethical restrictions.
