# Supplementary material for: Validation of the “Patient‐Acceptable Symptom State” Question as Outcome Measure in AChR Myasthenia Gravis: A Multicentre, Prospective Study
Source: Eur J Neurol. 2025 Jun 25;32(6):e70262. doi: 10.1111/ene.70262 (PMC12188101; doi:10.1111/ene.70262)
Supplement: Supplementary file 2 — Table S1. Comparison of the patient characteristics from the index and validation cohort. Table S2. Threshold of MG clinical scales for the definition of favorable outcome according to the PASS and MGFA‐PIS identified in the index cohort. Table S3. Validation of the MG scales defined patient acceptable symptom state (PASS) thresholds in validation cohort. Table S4. Multivariable logistic regression models to evaluate the clinical predictors of PASS=YES. Table S5. MG scales items association with patient acceptable symptom state (PASS) = YES: multivariable logistic regression. Table S6 Analysis of the association of demographic and clinical factors with disease expression at study visit within the same PASS‐defined patient subgroup. [file ENE-32-e70262-s001.doc]

Supplements.

**List of contents**

1. **Supplementary methods**
2. **Supplementary Figure Legend**
3. **Supplementary Tables**
4. **Table S1 – Comparison of the patient characteristics from the index and validation cohort**
5. **Table S2 – Threshold of MG clinical scales for the definition of favourable outcome according to the PASS and MGFA-PIS identified in the index cohort**
6. **Table S3 –** **Validation of the MG scales defined patient acceptable symptom state (PASS) thresholds in validation cohort**
7. **Table S4 – Multivariable logistic regression models to evaluate the clinical predictors of PASS=YES**
8. **Table S5 –** **MG scales items association with patient acceptable symptom state (PASS)=YES: multivariable logistic regression**
9. **Table S6 – Analysis of the association of demographic and clinical factors with disease expression at study visit within the same PASS-defined patient subgroup**
10. **Appendix - Italian translation of the Patient-acceptable symptom state (PASS) question used in the study.**

**1. Supplementary methods**

**Patient acceptable symptom state (PASS) question translation to Italian**

Two independent study evaluators (Italian native speakers), aware of the clinical context, independently translated the original PASS question into Italian. The two translations were compared and merged into a single version through discussion, resolving discrepancies. Two additional study evaluators, blind to the original, translated the synthesized version back into English to check for concordance. Finally, all the authors of the paper reviewed all versions to check for semantic, idiomatic, experiential, and conceptual equivalence and created a pre-final version of the Italian PASS question that was first tested on a small sample (10 MG patients). This sample of patients were interviewed to assess if the question was clear and culturally appropriate. Based on feedback, the final version of the question was finalized.

**Logistic regression models building**

For the multivariable logistic regression model building, we used a parsimonious modeling strategy to address potential overfitting and improve model interpretability. Candidate variables for multivariable logistic regression were selected based on both univariate performance, evaluation of multicollinearity (using the Variance Inflation Factor (VIF)) to avoid redundancy, and a priori clinical relevance. We formally assessed multicollinearity among MG-ADL, QMG, and MG-QOL15r scores using the Variance Inflation Factor (VIF). All VIFs were <3, indicating moderate collinearity. To limit overfitting, and in line with the number of events in the PASS=NO group (~50 patients), we constrained the final multivariable models to a maximum of 5 parameters and we build three separate models, each with one clinical scale (model 1: MG-ADL, model 2: QMG, model 3: MG-QoL15r) plus the same four clinical variables selected as outlined below (MG subtype, MG duration, treatment, MG severity). Model selection was guided by Akaike Information Criterion (AIC) and pseudo R-squared values. To compare overall model fit and discrimination, we reported the area under the receiver operating characteristic curve (AUC) and used the Hosmer-Lemeshow test for goodness-of-fit. To evaluate and compare the discriminatory ability of the three logistic regression models, the areas under the ROC curves (AUCs) were compared using DeLong’s test. Separate models were also fit using individual MG scales to assess their independent contribution to PASS prediction.

**Missing Data Assessment**

The pattern and mechanism of missing data were evaluated prior to analysis. Little’s Missing Completely at Random (MCAR) test was conducted to assess whether the missing values could be considered missing completely at random. The test indicated that the missing data were MCAR (Number of obs=173, χ²(df) = 22.20 (27), p =0.727, supporting the assumption that the missingness was not systematically related to the observed or unobserved data (data missingness frequency in each model reported in Table S4: Model 1 (PASS-MG ADL), missing observations=1.2%; Model 2 (PASS-QMG), missing observations=0.6%; missing observations=6.4%). As a result, complete case analysis deemed appropriate (Schober P & Vetter TR, Anesth Analg. 2020 Oct 20;131(5):1419–1420). The analysis of the missing data for the models assessing the association between MG-ADL, QMG and MG-QOL15r items / item subgroups with PASS=YES showed similar results (number of obs=173, χ²(df) = 53.1483 (46), p =0.2181). However, the data missingness rates in these models were higher (Table S5, model 1: 18.5%; model 2: 17.9%; model 3: 24.3%). This substantially reduced the power of this analysis which should be considered exploratory.

**2****. Supplementary Figure Legend**

**Figure S1. MG clinical scale scores and MGFA-PIS.** In the index cohort, patients with an MGFA-PIS=MM-or-better had a lower median MG-ADL score [0 (IQR=0-1) vs 5 (IQR=2-8), p<0.0001] (**A**), QMG score (4 (IQR=1-6) vs 11 (IQR=9-15), p<0.0001] (**B**), and MG-QoL15r score (1 (IQR=0-4) vs 12.5 (IQR=2-20.5), p<0.0001] (**C**) compared to patients who did not achieve MM-or-better. Receiver operating characteristic (ROC) curve estimating the MGFA-PIS=MM-or-better thresholds for MG-ADL score (AUC= 0.91, 95%C.I. 0.86-0.97) (**D**), QMG score (AUC=0.95, 95%C.I. 0.90-0-99) (**E**), and MG-QoL15r score (AUC=0.82, 95%C.I. 0.73-0.91) (**F**) in the index cohort. MG-ADL= Myasthenia gravis activity of daily living score; MG-QoL15r= myasthenia gravis quality of life 15 revised score; PASS=patient acceptable symptom state; QMG= quantitative myasthenia gravis score**.**

**3. Supplementary Tables**

| **Table S1. Comparison of the patient characteristics from the index and validation cohort** | | | |
| --- | --- | --- | --- |
|  | Index cohort  (N=111) | Validation cohort  (N=62) | P |
| Median age at onset, IQR (years) | 46 (27-65) | 59 (43-67) | ***0.02*** |
| Female, n. (%) | 48 (43%) | 28 (45%) | 0.80 |
| Median disease duration, IQR (years) | 7.2 (2.7-15) | 6.4 (2.3-12.7) | 0.55 |
| ***MG subtype*** |  |  |  |
| EOMG | 36 (32%) | 15 (24%) | 0.29 |
| LOMG | 13 (12%) | 11 (18%) | 0.36 |
| VLOMG | 24 (22%) | 15 (24%) | 0.71 |
| TAMG | 38 (34%) | 21 (34%) | 0.99 |
| ***Maximum MGFA clinical classification*** |  |  | 0.08 |
| I | 14 (13%) | 8 (13%) | 0.99 |
| II | 26 (23%) | 26 (42%) | ***0.0152*** |
| III | 43 (39%) | 17 (27%) | 0.18 |
| IV | 15 (14%) | 5 (8%) | 0.33 |
| V | 13 (12%) | 6 (10%) | 0.80 |
| Bulbar involvement at max. disease severity, n (%) | 68 (61%) | 31 (50%) | 0.15 |
| ***MG clinical scales*** |  |  |  |
| Median MG-ADL score, IQR | 1 (0-4) | 2 (0-3) | 0.82 |
| Median QMG score, IQR | 6 (4-11) | 5 (2-9) | 0.44 |
| Median MG-QOL15r score, IQR | 3 (0-10) | 2 (0-6) | 0.38 |
| Comorbidities | 79 (71%) | 53 (90%) | ***0.02*** |
| ***Treatment*** |  |  |  |
| No IS | 23 (21%) | 9 (15%) | 0.41 |
| CS | 28 (25%) | 32 (52%) | ***0.0008*** |
| CS+1 IS | 45 (41%) | 14 (23%) | ***0.02*** |
| CS + 2 or more IS | 15 (14%) | 7 (11%) | 0.50 |
| Thymectomy | 63 (57%) | 34 (55%) | 0.87 |
| Treatment-related AEs | 39 (35%) | 28 (45%) | 0.19 |
| ***MGFA PIS*** |  |  |  |
| CSR | 5 (5%) | 9 (15%) | ***0.04*** |
| PR | 9 (8%) | 5 (8%) | 0.99 |
| MM | 57 (51%) | 28 (45%) | 0.52 |
| I | 21 (19%) | 12 (19%) | 0.99 |
| U | 3 (3%) | 2 (3%) | 0.99 |
| W | 7 (6%) | 5 (8%) | 0.76 |
| Not applicable (study visit occurred before therapy initiation) | 9 (8%) | 1 (2%) | 0.10 |
| MM-or-better | 70 (69%)^a^ | 42 (68%) | 0.91 |
| PASS = YES | 82 (74%) | 42 (68%) | 0.39 |

*AE: adverse event; CS: corticosteroids; CSR: complete stable remission; EOMG: early-onset myasthenia gravis; I: improved; IQR: interquartile range; IS: immunosuppressant; LOMG: late-onset myasthenia gravis; MG: myasthenia gravis; MG-ADL: myasthenia gravis activity of daily living scale; MGFA: Myasthenia Gravis Foundation of America; MG-QOL15r: myasthenia gravis quality of life 15-revised; MM: minimal manifestations; PASS= patient-acceptable symptom state; PR: pharmacological remission; QMG: quantitative myasthenia gravis scale; TAMG: thymoma-associated myasthenia gravis; U: unchanged; VLOMG: very-late onset myasthenia gravis; W: worsened.*

| **Table S2. Threshold of MG clinical scales for the definition of favourable outcome according to the PASS and MGFA-PIS identified in the index cohort** | | | | | | | | |
| --- | --- | --- | --- | --- | --- | --- | --- | --- |
| **Optimal PASS=YES cut-offs** | | | | | | | | |
|  | Threshold | AUC  (95% CI) | Sensitivity  (95% CI) | Specificity  (95% CI) | P | Accuracy | PPV | NPV |
| MG ADL | ≤2 | 0.92  (0.86-0.97) | 89.2%  (80.7-94.2%) | 92.9%  (77.4-98.7%) | <0.0001 | 90.2% | 97.3% | 75.2% |
| QMG | ≤8 | 0.85  (0.77-0.93) | 84.2%  (74.7-90.5%) | 85.2%  (67.5-94.1%) | <0.0001 | 84.5% | 94.2% | 65.6% |
| MG QOL15r | ≤6 | 0.92  (0.80-0.95) | 89.0  (80.4-94.1%) | 86.2%  (69.4%-94.5%) | <0.0001 | 88.3% | 94.8% | 73.5% |
| **Optimal MGFA=MM-or-better cut-offs** | | | | | | | | |
|  | Threshold | AUC  (95% CI) | Sensitivity  (95% CI) | Specificity  (95% CI) | P | Accuracy | PPV | NPV |
| MG ADL | ≤2 | 0.91  (0.86-0.97) | 92.9%  (84.3-96.9%) | 71.2%  (54.6-84.4%) | <0.0001 | 86.1% | 87.5% | 82.1% |
| QMG | ≤7 | 0.95  (0.90-0.99) | 88.6%  (79.0-94.1%) | 90.0%  (74.4-96.5%) | <0.0001 | 89.0% | 95.1% | 78.3% |
| MG-QOL15r | ≤9 | 0.82  (0.73-0.91) | 94.3%  (86.2-97.7%) | 59.4%  (42.3-74.5%) | <0.0001 | 83.4% | 83.5% | 82.7% |

*The table shows the thresholds of the MG-ADL, QMG and MG-QoL15r best defining PASS=YES and MGFA-PIS=MM-or-better (thresholds selected based on the highest Youden’s Index). The area under the curve and the P value are calculated with the ROC curve analysis. Sensitivity, specificity, accuracy, positive and negative predictive values associated with each thresholds are also shown. AUC= area under the curve; CI=confidence interval; MG-ADL=Myasthenia gravis activity of daily living score; MG-QoL15r= myasthenia gravis quality of life 15 revised score; MM-or-better= minimal manifestation-or-better; PASS= patient acceptable symptom state; PPV= positive predictive value; NPV=negative predictive value; QMG= quantitative myasthenia gravis score.*

| **Table S3. Validation of the MG scales defined patient acceptable symptom state (PASS) thresholds in validation cohort** | | | |
| --- | --- | --- | --- |
|  | **MG ADL-anchored PASS = YES** | **MG ADL-anchored PASS = NO** | **P** |
| PASS=YES^a^, n(%) | 31/37 (84%) | 10/23 (44%) | ***0.001*** |
| MGFA PIS= MM-or-better, n (%) | 29/37 (78%) | 12/23 (52%) | ***0.034*** |
|  | **QMG-anchored PASS = YES** | **QMG-anchored PASS = NO** | **P** |
| PASS=YES^a^, n(%) | 36/45 (80%) | 6/17 (35%) | ***0.001*** |
| MGFA PIS= MM-or-better, n (%) | 34/42 (80%) | 7/19 (35%) | ***0.001*** |
|  | **MG-QOL15r-anchored PASS = YES** | **MG-QOL15r-anchored PASS = NO** | **P** |
| PASS=YES^a^, n(%) | 32/40 (80%) | 4/11 (36%) | ***0.009*** |
| MGFA PIS= MM-or-better, n (%) | 35/44 (83%) | 3/7 (43%) | 0.06 |

*a: actual response to the PASS question provided from the patients of the validation cohort; Note: MG-ADL was not available in 2 patients; MG-QoL15r was not available in 11 patients.*

*MG: myasthenia gravis; MG-ADL: myasthenia gravis activity of daily living scale; MGFA: Myasthenia Gravis Foundation of America; MG-QOL15r: myasthenia gravis quality of life 15-revised; MM: minimal manifestations; PASS= patient-acceptable symptom state;*

| **Table S4. Multivariable logistic regression models to evaluate the clinical predictors of PASS=YES** | | |
| --- | --- | --- |
| Model [n. of observations included and goodness of fit parameters] / Independent variable | OR (95% CI) | P |
| **Model 1 [N=171, Pseudo-R^2^= 0.45; AIC=128.1; Hosmer–Lemeshow ꭕ^2^ = 4.05 (p=0.85); AUC=0.91]** | | |
| MG-ADL score (1-point increase) | 0.46 (0.36-0.60) | ***<0.001*** |
| Disease duration (years) | 1.02 (0.96-1.07) | 0.585 |
| ***MG subtype^a^*** |  |  |
| EOMG | (ref) | - |
| LOMG | 6.30 (0.89-44.43) | 0.065 |
| VLOMG | 1.68 (0.33-8.49) | 0.533 |
| Thymoma-associated MG | 0.60 (0.16-2.24) | 0.452 |
| Moderate MG to MG crisis (MGFA: III-V) at maximum disease severity | 1.11 (0.35-3.50) | 0.864 |
| Treatment^b^ | 1.34 (0.72-2.50) | 0.355 |
| **Model 2 [N=172, Pseudo-R^2^= 0.36; AIC=145.9; Hosmer–Lemeshow ꭕ^2^ = 2.52 (p=0.96); AUC=0.87]** | | |
| QMG score (1-point increase) | 0.72 (0.64-0.81) | ***<0.001*** |
| Disease duration (years) | 1.02 (0.97-1.08) | 0.35 |
| ***MG subtype^a^*** |  |  |
| EOMG | (ref) | - |
| LOMG | 4.33 (0.62-30.2) | 0.139 |
| VLOMG | 1.37 (0.31-6.07) | 0.678 |
| Thymoma-associated MG | 0.75 (0.23-2.51) | 0.642 |
| Moderate MG to MG crisis (MGFA: III-V) at maximum disease severity | 1.24 (0.39-3.95) | 0.712 |
| Treatment^b^ | 1.24 (0.71-2.18) | 0.456 |
| **Model 3 [N=162, Pseudo-R^2^= 0.40; AIC=129.6; Hosmer–Lemeshow ꭕ^2^ = 4.79 (p=0.78); AUC=0.88]** | | |
| MG-QOL15r score (1-point increase) | 0.76 (0.70-0.84) | <0.001 |
| Disease duration (years) | 1.01 (0.95-1.07) | 0.832 |
| ***MG subtype^a^*** |  |  |
| EOMG | (ref) | - |
| LOMG | 3.26 (0.50-21.50) | 0.219 |
| VLOMG | 1.84 (0.36-9.37) | 0.463 |
| Thymoma-associated MG | 0.83 (0.23-2.99) | 0.774 |
| Moderate MG to MG crisis (MGFA: III-V) at maximum disease severity | 0.97 (0.31-3.01) | 0.953 |
| Treatment^b^ | 1.39 (0.78-2.49) | 0.266 |

Notes: a. Age at onset was not included in the multivariable logistic analysis model, as this information is already included in the “MG subtype” classification; for the multivariable analysis, the “MG Subtype” variable was subdivided in the following categories: thymoma-associated MG (any age), or, in non-thymoma MG, early-onset MG (<50 y), late-onset MG (50-64 y), very late-onset MG (≥65 y); the early-onset MG class was used as reference b: Treatment burden was classified as shown in Table 1, based on the exposure to corticosteroids and immunosuppressants: (1) no steroids or immunosuppressants (IS) (“no IS”), (2) only corticosteroids (“CS”), (3) CS + 1 IS; (4) CS + ≥2 IS.

| **Table S5. MG scales items association with patient acceptable symptom state (PASS)=YES: multivariable logistic regression** | | |
| --- | --- | --- |
| Independent variable | OR (95% CI) | P |
| ***Model 1: MG ADL [N=141,* Pseudo-R^2^= 0.16; AIC=148.4; Hosmer–Lemeshow ꭕ^2^ = 2.17 (p=0.83); AUC=0.74]** | | |
| Ocular items | 0.46 (0.31-0.67) | ***<0.001*** |
| Limb items | 0.89 (0.59-1.37) | 0.619 |
| Bulbar items | 1.02 (0.74-1.40) | 0.91 |
| ***Model 2: QMG [N=142,* Pseudo-R^2^= 0.13; AIC=151.7; Hosmer–Lemeshow ꭕ^2^ = 2.68 (p=0.91); AUC=0.71]** | | |
| Ocular items | 0.65 (0.51-0.84) | ***0.001*** |
| Limb items | 0.96 (0.80-1.15) | 0.657 |
| Bulbar items | 0.78 (0.57-1.07) | 0.128 |
| Neck | 0.87 (0.40-1.92) | 0.732 |
| ***Model 3: MG-QOL15r[N=131,* Pseudo-R^2^= 0.19; AIC=155.9; Hosmer–Lemeshow ꭕ^2^ = 4.54 (p=0.60); AUC=0.75]** | | |
| Q1: I am frustrated by my MG | 0.59 (0.20-1.76) | 0.344 |
| Q2: I have trouble with my eyes because of my MG (e.g. double vision) | 0.30 (0.13-0.67) | ***0.003*** |
| Q3: I have trouble eating because of MG | 0.46 (0.12-1.77) | 0.257 |
| Q4: I have limited my social activity because of my MG | 0.61 (0.19-1.96) | 0.41 |
| Q5: My MG limits my ability to enjoy hobbies and fun activities | 1.46 (0.40-5.28) | 0.566 |
| Q6: I have trouble meeting the needs of my family because of my MG | 1.35 (0.47-3.87) | 0.578 |
| Q7: I have to make plans around my MG | 1.04 (0.31-3.54) | 0.947 |
| Q8: I am bothered by limitations in performing my work (include work at home) because of my MG | 0.64 (0.25-1.65) | 0.351 |
| Q9: I have difficulty speaking due to MG | 1.79 (0.53-6.01) | 0.346 |
| Q10: I have lost some personal independence because of my MG (e.g. driving, shopping, running errands) | 0.51 (0.16-1.57) | 0.241 |
| Q11: I am depressed about my MG | 1.20 (0.33-4.39) | 0.788 |
| Q12: I have trouble walking due to MG | 1.19 (0.37-3.85) | 0.773 |
| Q13: I have trouble getting around public places because of my MG | 1.41 (0.29-6.82) | 0.67 |
| Q14: I feel overwhelmed by my MG | 1.16 (0.19-7.25) | 0.872 |
| Q15: I have trouble performing my personal grooming needs due to MG | 1.49 (0.21-10.52) | 0.692 |

*MG: myasthenia gravis MG-ADL: myasthenia gravis activity of daily living scale; MG-QOL15r: myasthenia gravis quality of life 15-revised; QMG: quantitative myasthenia gravis scale.*

| **Table S6. Analysis of the association of demographic and clinical factors with disease expression at study visit within the same PASS-defined patient subgroup** | | | |
| --- | --- | --- | --- |
| **Subgroup:** **patient acceptable symptom state (PASS)=YES** | | | |
|  | **Age at onset <50 years** | **Age at onset ≥50 years** | **P** |
| Median MG-ADL score (IQR) | 0 (0-2) | 1 (0-2) | 0.0982 |
| Median QMG score (IQR) | 5 (1-8) | 4 (2-6) | 0.8527 |
| Median MG-QOL15r score (IQR) | 1 (0-4) | 1 (0-3) | 0.8464 |
|  | **Sex: female** | **Sex: male** | **P** |
| Median MG-ADL score (IQR) | 0 (0-2) | 1 (0-2) | 0.1873 |
| Median QMG score (IQR) | 5 (2-8) | 4 (2-6) | 0.3284 |
| Median MG QOL score (IQR) | 2 (0-3) | 1 (0-3) | 0.6910 |
|  | **Max. MGFA=II** | **Max.MGFA=III-V** | **P** |
| Median MG-ADL score (IQR) | 1 (0-2) | 1 (0-2) | 0.4754 |
| Median QMG score (IQR) | 4 (2-6) | 5 (3-8) | ***0.022*** |
| Median MG QOL score (IQR) | 1 (0-3) | 2 (0-5) | 0.5418 |
| **Subgroup: patient acceptable symptom state (PASS)=NO** | | | |
|  | **Age <50** | **Age >50** | **P** |
| Median MG-ADL score (IQR) | 5 (3.5-9) | 4 (3-8) | 0.3356 |
| Median QMG score (IQR) | 11 (9-16) | 11 (6-15) | 0.3912 |
| Median MG QOL score (IQR) | 14 (8-21) | 9 (4-18.5) | 0.2055 |
|  | **Sex: female** | **Sex: male** | **P** |
| Median MG-ADL score (IQR) | 6.5 (3-9) | 4 (3-6) | 0.232 |
| Median QMG score (IQR) | 12 (9-19) | 10.5 (8-13) | 0.118 |
| Median MG QOL score (IQR) | 14 (7-19) | 12 (3.5-21) | 0.6372 |
|  | **Max. MGFA=II** | **Max.MGFA=III-V** | **P** |
| Median MG-ADL score (IQR) | 3.5 (2-4.5) | 6.5 (4-9) | ***0.0115*** |
| Median QMG score (IQR) | 7 (2-11) | 12 (10-19) | ***0.0059*** |
| Median MG QOL score (IQR) | 9 (4-18.5) | 14.5 (8.5-20.5) | 0.3124 |

*IQR: interquartile range; MG-ADL: myasthenia gravis activity of daily living scale; MG-QOL15r: myasthenia gravis quality of life 15-revised; QMG: quantitative myasthenia gravis scale.*

**4. Appendix**

**Italian translation of the Patient-acceptable symptom state (PASS) question used in the study.**

Original PASS question (Mendoza M et al., Neurology 2020; manuscript ref.#5): “Considering all the ways you are affected by Myasthenia, if you had to stay in your current state for the next months, would you say that your current disease state status is satisfactory?”

Italian translation of the PASS question: “Considerando globalmente l’impatto che la miastenia gravis ha su di Lei, se dovesse rimanere nelle attuali condizioni di salute per i prossimi mesi, sarebbe soddisfatto del compenso raggiunto?”
